# Spatiotemporal Risk Assessment of H5 Avian Influenza in China: An Interpretable Machine Learning Approach to Uncover Multi-Scale Drivers

**DOI:** 10.3390/ani15162447

**Published:** 2025-08-20

**Authors:** Xinyi Wang, Yihui Xu, Xi Xi

**Affiliations:** 1 College of Veterinary Medicine, China Agricultural University, No. 2 Yuanmingyuan West Road, Haidian District, Beijing 100193, China; xinyi.wang@cau.edu.cn (X.W.); xyh@cau.edu.cn (Y.X.); 2 China Agricultural University, No. 2 Yuanmingyuan West Road, Haidian District, Beijing 100193, China

**Keywords:** H5 avian influenza, spatiotemporal modeling, machine learning, ecological drivers, risk mapping

## Abstract

Avian influenza (AI), particularly the H5 subtypes, poses a significant and persistent threat globally. While the influence of environmental factors on AI seasonality is recognized, a comprehensive understanding of the hierarchical and interactive effects of multi-scale drivers in a vast and ecologically diverse country like China remains limited. We developed an interpretable machine learning framework (XGBoost with SHAP) to analyze the spatiotemporal risk of 1800 H5 AI outbreaks in mainland China from 2000 to 2023. We integrated multi-source data, including dynamic poultry density, Köppen climate classifications, Important Bird and Biodiversity Areas (IBAs), and daily meteorological variables, to identify key drivers and quantify their nonlinear and synergistic effects. The model demonstrated high predictive accuracy (5-fold cross-validation $R^{2}$ = 0.776). Our analysis revealed that macro-scale ecological contexts, particularly poultry density and specific Köppen climate zones (e.g., Cwa), and strong seasonality were the most dominant drivers of AI risk. We identified significant nonlinear relationships, such as a strong inverse relationship with temperature, and a critical synergistic interaction where high temperatures substantially amplified risk in areas with high poultry density. The final predictive map identified high-risk hotspots primarily concentrated in eastern and southern China. Our findings indicate that H5 AI risk is governed by a hierarchical interplay of multi-scale environmental drivers. This interpretable modeling approach provides a valuable tool for developing targeted surveillance and early warning systems to mitigate the threat of avian influenza.

## Simple Summary

Avian influenza, particularly the H5 subtypes, poses a continuous threat to poultry farming and public health. Predicting where and when outbreaks might occur is difficult, especially in a large and diverse country like China. To tackle this challenge, we developed a smart computer model using artificial intelligence (XGBoost) to analyze over two decades of H5 avian influenza outbreak data. We combined this with multi-source information, including satellite data on poultry density, climate zones, wild bird habitats, and daily weather conditions. Our model successfully identified the key factors driving outbreak risk with high accuracy. We found that the density of poultry farms and specific climate zones are the most important background factors, while daily weather changes act as triggers. Notably, we discovered a surprising interaction: hot summer temperatures, usually considered low-risk, can become a significant danger in areas with many poultry farms. These findings were used to create a high-resolution risk map of China, highlighting hotspots for targeted surveillance. This research provides a valuable tool for developing better early warning systems to protect both animal and human health from the threat of avian influenza.

## 1. Introduction

Avian influenza (AI) viruses, particularly highly pathogenic (HPAI) H5 subtypes, represent a persistent threat to global food security, wildlife conservation, and public health [1]. Originating from a vast gene pool in wild aquatic birds, these viruses can cause devastating outbreaks in poultry populations, leading to substantial economic losses. In recent years, the scale of highly pathogenic avian influenza (HPAI) epidemics has become unprecedented. The 2021–2022 H5N1 wave, for instance, resulted in over 6227 cases across 37 European countries, surpassing all previous outbreaks in the region. This escalating situation, coupled with sporadic but severe zoonotic infections in humans, has intensified concerns regarding their pandemic potential. The ecology of these viruses is deeply intertwined with their natural reservoirs, domestic poultry systems, and a range of environmental factors [2,3]; therefore, elucidating the drivers of AI transmission remains a critical global priority.

The epidemiology of HPAI is governed by a confluence of drivers operating at multiple scales. The density of poultry populations, as the primary host, is a recognized determinant of risk at both regional and global scales. Likewise, meteorological variables are known to influence viral viability, while wild birds serve as critical reservoirs and dispersal vectors. Despite this knowledge, a robust predictive understanding of H5 AI risk is constrained by several persistent methodological and conceptual limitations [4].

First, the prevalent use of statistical models assuming linearity often fails to capture the complex, nonlinear, and threshold-dependent responses inherent in ecological systems. To overcome these limitations, machine learning and deep learning approaches are increasingly being adopted across various agricultural and ecological domains, from crop monitoring to disease detection [5,6,7]. Second, large-scale analyses have frequently been limited by a reliance on static data, thereby neglecting the dynamic nature of key anthropogenic factors such as poultry production. Most critically, the hierarchical and interactive effects among drivers remain insufficiently characterized [8]. The mechanisms by which stable, macro-scale contexts (e.g., climate zones) modulate the impact of transient, micro-scale triggers (e.g., daily weather fluctuations) are not yet fully elucidated [9,10,11]. A framework capable of simultaneously addressing these challenges is therefore essential for advancing predictive capabilities.

Prior risk-mapping studies in China have already linked HPAI circulation to agro-ecological contexts. In particular, Martin et al. [12] used boosted regression trees and logistic regression to show that clinical outbreaks were mainly associated with chicken density, human population density, and low elevation, whereas risk detected through active surveillance related more strongly to domestic duck density and surface water. Building on this foundational work, our study extends the temporal coverage to 2000–2023, incorporates dynamic (rather than static) poultry density data and detailed Köppen climate classifications, and leverages the XGBoost-SHAP framework to explicitly quantify the nonlinear effects and cross-scale interactions that were previously difficult to interpret.

To address these multifaceted challenges, this study develops and applies an interpretable machine learning framework to comprehensively investigate the drivers of H5 avian influenza risk in China, using over two decades of dynamic, multi-source data. The specific objectives are threefold:

1. To employ a powerful gradient boosting algorithm (XGBoost) [13] to capture complex, nonlinear driver responses and elucidate the hierarchical structure of risk, distinguishing between foundational macro-scale contexts and transient micro-scale triggers;
2. To leverage the SHAP (SHapley Additive exPlanations) framework to explicitly quantify the magnitude and form of both nonlinear relationships and synergistic interactions among key drivers;
3. To synthesize these complex model insights into a practical, high-resolution national risk map to inform targeted interventions.

Through this integrated approach, this study seeks to provide a more nuanced and holistic understanding of H5 AI epidemiology, offering valuable insights for the development of evidence-based surveillance and early warning systems.

The remainder of this paper is structured as follows. Section 2 details the data sources and our interpretable machine learning methodology. Section 3 presents the model’s predictive performance and the key findings regarding the hierarchical, nonlinear, and synergistic nature of the risk drivers. Section 4 discusses the advantages, limitations, and future perspectives of our study, and Section 5 concludes this paper.

## 2. Materials and Methods

### 2.1. Avian Influenza Outbreak Data

Our study is based on outbreak data for highly pathogenic avian influenza (HPAI) H5 subtypes in mainland China, obtained from the Food and Agriculture Organization (FAO)’s Global Animal Disease Information System (EMPRES-i). The dataset encompasses all reported events from 25 November 2003 to 11 May 2024. We focused specifically on HPAI H5 due to its significant impact on poultry production and public health.

The integrity of these data is fundamentally underpinned by the international surveillance framework of the World Organisation for Animal Health (WOAH, formerly OIE). As a WOAH-listed disease, the confirmation and reporting of avian influenza follow stringent, standardized procedures. Case confirmation adheres to scientific standards outlined in the WOAH’s Manual of Diagnostic Tests and Vaccines for Terrestrial Animals, which recommends molecular techniques like real-time RT-PCR for definitive virus detection [14]. Once confirmed, member countries are obligated to report outbreaks under the Terrestrial Animal Health Code [15]. This rigorous validation process ensures that the data used in our study are of high quality.

To construct our final analytical dataset, the raw data underwent a multi-step preprocessing pipeline. First, we spatially filtered the records to retain only those within mainland China and temporally selected events up to 11 May 2024. This resulted in a dataset exclusively comprising the following serotypes: H5N1, H5N2, H5N3, H5N6, and H5N8 HPAI. We then aggregated these closely related subtypes to model the overall HPAI H5 risk landscape. An outbreak event was defined as one or more confirmed cases at a unique location on a specific day; accordingly, case counts for reports sharing the same coordinates and date were summed. The “observation date” was used as the primary temporal marker for each event, as it most closely reflects the timing of field detection. This process yielded a final dataset of 1800 distinct outbreak events, with the aggregated case count serving as the model’s target variable.

### 2.2. Environmental and Ecological Predictors

A comprehensive set of potential predictors was constructed by integrating multi-source geospatial and meteorological datasets, a common practice in modern ecological studies that increasingly leverages advanced remote sensing technologies for data acquisition [16,17]. All data layers were standardized to a consistent spatial resolution and projected to the WGS 84 coordinate system.

#### 2.2.1. Meteorological Data

Daily meteorological data were sourced from the China Meteorological Administration (CMA), ensuring consistency and authority across the entire study period. To achieve complete temporal coverage, we constructed a continuous time-series dataset by integrating two official sources from the CMA.

For the period prior to 2014, we utilized the China National-Level Ground Meteorological Station Basic Meteorological Elements Daily Value Dataset (V3.0), provided by the CMA’s National Meteorological Information Center [18]. This foundational dataset is exceptionally reliable, as documented in its official evaluation report, which confirms that its data availability rate exceeds 99% and its accuracy rate approaches 100% following a rigorous, multi-stage quality control process [18]. For the period from 2014 onward, where a consolidated V3.0-style dataset was not yet available, we obtained data directly from the CMA’s operational data sharing service. This service provides real-time and recent historical data from the same network of national-level ground stations, ensuring methodological continuity and data compatibility with the earlier V3.0 dataset.

The final variables included daily mean temperature (°C), daily mean atmospheric pressure (hPa), and cumulative daily precipitation (mm). To ensure that these data accurately reflected local conditions, a boundary-based matching approach was employed. Each outbreak point was first located within its respective smallest administrative unit (e.g., county or district), and subsequently, data from the official weather station operating within that same administrative unit were assigned to the point. To capture potential lagged effects, data were extracted for both the outbreak date and the three preceding days.

#### 2.2.2. Poultry Density Data

To account for the distribution and density of primary domestic hosts, a critical driver of AI transmission, we incorporated dynamic poultry density data from the Gridded Livestock of the World (GLW), a globally recognized, peer-reviewed dataset developed by the Food and Agriculture Organization (FAO) [19,20]. The GLW provides standardized, high-resolution (approx. 10 km) estimates of livestock populations, generated using a sophisticated Random Forest modeling approach based on the most recently compiled subnational census data [20]. This makes it a highly suitable and authoritative source for our large-scale analysis. We specifically focused on the combined density of chickens and ducks, as they are the most significant poultry species implicated in the epidemiology of H5 avian influenza in China [19].

To capture the spatiotemporal evolution of poultry farming across our long study period, we utilized the available GLW raster layers for the years 2010, 2015, and 2020. A dynamic temporal matching approach was employed: each outbreak event was matched to the most temporally proximate GLW map. For instance, an outbreak occurring between 2010 and 2012 was matched to the 2010 map, while an outbreak in 2018 was matched to the 2020 map. This method represents a significant improvement over using a single, static host distribution map, as it better reflects the changing landscape of poultry production over time.

For each matched outbreak location, the corresponding poultry density value (heads/birds per km^2^) was extracted. Given that poultry density data are often characterized by a highly skewed distribution, a log-plus-one transformation (log(x+1)) was applied. This standard procedure normalizes the variable’s distribution and stabilizes its variance, thereby improving its performance and interpretability within the machine learning model.

#### 2.2.3. Köppen–Geiger Climate Classification Data

To characterize the macro-climatic context of each outbreak, a high-resolution (1 km) Köppen–Geiger climate classification map was utilized [21]. The specific dataset employed was the latest version released in 2023, which provides climate zone projections for 1901–2099 based on constrained CMIP6 data. Each outbreak coordinate was spatially joined with these climate polygons to assign a specific classification code (e.g., “Dwa”, “Cfa”), thereby embedding the long-term climatic background as a categorical predictor in the model.

#### 2.2.4. Important Bird and Biodiversity Area (IBA) Data

The potential influence of wild bird reservoirs was represented using geospatial data of Important Bird and Biodiversity Areas (IBAs) from BirdLife International (version updated March 2025). Two distinct features were engineered from this dataset:

1. A binary variable was created to indicate whether an outbreak occurred directly within an IBA polygon;
2. For outbreaks located outside these zones, the Euclidean distance (in kilometers) to the boundary of the nearest IBA was calculated.

### 2.3. Feature Engineering and Selection

A comprehensive set of predictors was engineered, grounded in established biological and ecological principles of AI transmission. The cornerstone of our feature engineering was the creation of a biologically informed temporal structure. A substantial body of evidence indicates that the typical incubation period of HPAI H5 viruses in poultry ranges from 1 to 3 days [14]. Based on this critical window of infection, we expanded each outbreak event into three parallel records representing the meteorological conditions at 1, 2, and 3 days prior. This fixed-lag approach was deliberately chosen for its direct biological interpretability. While more complex methods like Distributed Lag Nonlinear Models (DLNMs) exist for exploring a wider range of lag–response relationships [22], our focused 1–3 day window provides a clear and targeted test of the hypothesis that short-term environmental triggers during the incubation period are key drivers of outbreaks. A suite of features was derived from this structure, including lagged weather variables, weather dynamics indicators, and spatiotemporal controls, to capture a variety of environmental pressures. The justification for including these variable categories is detailed in Table 1.

This entire feature engineering process is formally summarized by Equation (1):

(1) $$x_{i,t-k}=\mathrm{Env}\left(s_{i},t_{i}-k\right),\;k=1,2,3$$

Here, $x_{i,t-k}$ represents the complete feature vector for the *i*-th outbreak, which occurred at location $s_{i}$ and time $t_{i}$. The vector captures the environmental conditions lagged by *k* days. The function Env(s,t) generates the full set of environmental predictors for a given location *s* and time *t*. As specified, the lag variable *k* covers the 1, 2, and 3 days corresponding to the viral incubation period.

Following feature engineering, a multi-stage feature selection process was implemented to derive a parsimonious and mechanistically interpretable model. This process was designed to systematically reduce dimensionality while retaining predictors with the most significant and biologically plausible influence. These predictors include three main categories: foundational lagged variables Equation (2), a suite of weather dynamics indicators Equation (3), and cyclical temporal controls Equation (4). The pipeline began with an ecological stratification of all predictors into macro-, meso-, and micro-scale categories to delineate their hierarchical scales of influence. Subsequently, an ensemble ranking approach was employed to robustly evaluate feature importance and mitigate the bias of any single algorithm. This ensemble combined machine learning-based importance (Random Forest) to capture nonlinear contributions with a traditional statistical test (Spearman’s rank correlation, p<0.05) to assess monotonic relationships. The final subset of 26 predictors was determined by integrating these rankings with domain knowledge on biological plausibility. The robustness of this feature set was further confirmed through a bootstrap stability assessment, ensuring the selected variables possess not only statistical significance but also plausible mechanistic links to AI transmission.

The specific mathematical definitions for the engineered feature categories are as follows. The foundational lagged variables, representing the mean temperature (*T*, in °C), mean atmospheric pressure (*P*, in hPa), and cumulative precipitation (*R*, in mm), are given by

(2) $$\left\{T_{t-k},P_{t-k},R_{t-k}\right\},\;k\in \left\{1,2,3\right\}$$

The weather dynamics and stability indicators were defined for any meteorological variable X∈{T,P,R}.

The cyclical temporal controls were defined for month $m_{i}$ and day of year $d_{i}$:

(3a) $$\begin{array}{cc} \Delta X_{t} & =X_{t}-X_{t-1} \end{array}$$

(3b) $$\begin{array}{cc} S_{t}^{X} & =\frac{1}{1+|\Delta X_{t}|} \end{array}$$

(3c) $$\begin{array}{cc} {\bar{X}}_{t}^{\left(3\right)} & =\frac{1}{3}\sum_{j=0}^{2}X_{t-j} \end{array}$$

The cyclical temporal controls were defined for month $m_{i}$ and day of year $d_{i}$:

(4a) $$\begin{array}{cc} {\mathrm{MonthSin}}_{i} & =\sin\;\left(\frac{2\pi m_{i}}{12}\right) \end{array}$$

(4b) $$\begin{array}{cc} {\mathrm{MonthCos}}_{i} & =\cos\;\left(\frac{2\pi m_{i}}{12}\right) \end{array}$$

(4c) $$\begin{array}{cc} {\mathrm{DOYSin}}_{i} & =\sin\;\left(\frac{2\pi d_{i}}{365}\right) \end{array}$$

(4d) $$\begin{array}{cc} {\mathrm{DOYCos}}_{i} & =\cos\;\left(\frac{2\pi d_{i}}{365}\right) \end{array}$$

### 2.4. Interpretable Machine Learning Model

To model the complex, nonlinear relationships between the multi-scale predictors and HPAI H5 outbreak counts, we employed XGBoost (Extreme Gradient Boosting), a powerful and widely used machine learning algorithm [13]. The utility of such advanced algorithms has been demonstrated in a wide range of smart agriculture applications, tackling complex tasks like phenotype analysis and automated weed control [27,28]. XGBoost is an ensemble method that builds a predictive model in the form of a collection of decision trees, which are added sequentially to correct the errors of the previous ones. This gradient boosting framework is highly effective at capturing complex interactions and nonlinear patterns without prior assumptions about the underlying functional form.

Our modeling approach is rooted in the principles of a Generalized Linear Model (GLM), specifically a Poisson regression, which is suitable for count data like outbreak events. The model links the set of predictors for the *i*-th observation, $x_{i}$, to the expected outbreak count, $\lambda_{i}$, via a logarithmic link function. This relationship is expressed as

(5) $$\ln\left(\lambda_{i}\right)=f\left(x_{i}\right)\;\mathrm{or}\;\mathrm{equivalently}\;\lambda_{i}=\exp\left(f\left(x_{i}\right)\right)$$

Here, the crucial difference from a standard GLM is that $f(x_{i})$ is not a simple linear combination of predictors. Instead, $f(x_{i})$ represents the complex, nonlinear function learned by the ensemble of XGBoost trees. It is the sum of the predictions from all the individual trees in the model.

To validate the choice of XGBoost, its performance was benchmarked against two traditional statistical models: a Panel Data model with fixed effects (PanelOLS) and a Gaussian Generalized Linear Model (GLM). All models were trained and evaluated on the same set of predictors using an identical 5-fold cross-validation scheme to ensure a fair comparison. As shown in Table 2, the XGBoost model achieved a mean $R^{2}$ of 0.776, substantially outperforming both the PanelOLS ($R^{2}$ = 0.458) and the GLM ($R^{2}$ = 0.257).This significant performance gap underscores the necessity of a machine learning approach capable of capturing the complex nonlinearities and interactions inherent in the ecological drivers of avian influenza, which traditional models fail to adequately address.

To interpret the outputs of this model and understand the contribution of each predictor to $f(x_{i})$, we integrated the SHAP (SHapley Additive exPlanations) framework [29]. All data processing, modeling, and visualization were conducted using Python (version 3.9.0). The machine learning model was implemented with the XGBoost library (version 2.1.4), and model interpretation was performed using the SHAP library (version 0.48.0). Key libraries for data manipulation and analysis included Pandas (version 2.2.3), NumPy (version 1.23.0), and Scikit-learn (version 1.6.1). Geospatial data were handled with GeoPandas (version 1.0.1), and figures were generated using Matplotlib (version 3.9.4) and Seaborn (version 0.13.2).

### 2.5. Model Validation and Diagnostics

The generalization ability and predictive performance of the final XGBoost model were rigorously evaluated using a 5-fold cross-validation (CV) scheme. We employed a spatial partitioning strategy for the folds to provide a more robust assessment of the model’s capacity to generalize across the diverse geographical contexts of the study area. Model performance was quantified using three standard metrics: the coefficient of determination ($R^{2}$), root mean square error (RMSE), and mean absolute error (MAE). The optimal set of model hyperparameters was selected through a systematic tuning process that aimed to maximize the cross-validated $R^{2}$ score. The final optimized hyperparameters are detailed in Appendix A.

In addition to performance evaluation, a suite of diagnostic tests was conducted to ensure model stability and integrity. To assess multicollinearity among the predictors, we calculated the Variance Inflation Factor (VIF). To test for spatial autocorrelation in the model’s residuals, which could indicate unexplained spatial patterns, we calculated Moran’s I statistic. The standard formulas for these diagnostics are given in Equation (6):

(6a) $$\begin{array}{cc} {\mathrm{VIF}}_{j} & =\frac{1}{1-R_{j}^{2}} \end{array}$$

(6b) $$\begin{array}{cc} I & =\frac{N}{W}\;\cdot \;\frac{\sum_{i}\sum_{j}w_{ij}\left(e_{i}-\bar{e}\right)\left(e_{j}-\bar{e}\right)}{\sum_{i}{\left(e_{i}-\bar{e}\right)}^{2}} \end{array}$$

In these diagnostics, $R_{j}^{2}$ is the coefficient of determination from regressing predictor *j* on all other predictors [30]. For Moran’s I, *N* is the number of spatial units, $e_{i}$ is the residual for unit *i*, $\bar{e}$ is the mean of the residuals, $w_{ij}$ is the spatial weight between units *i* and *j*, and *W* is the sum of all weights [31]. These diagnostics confirmed the statistical robustness of our final model.

## 3. Results

### 3.1. Descriptive Analysis: Spatiotemporal Overview of Outbreaks

**Figure 1:**
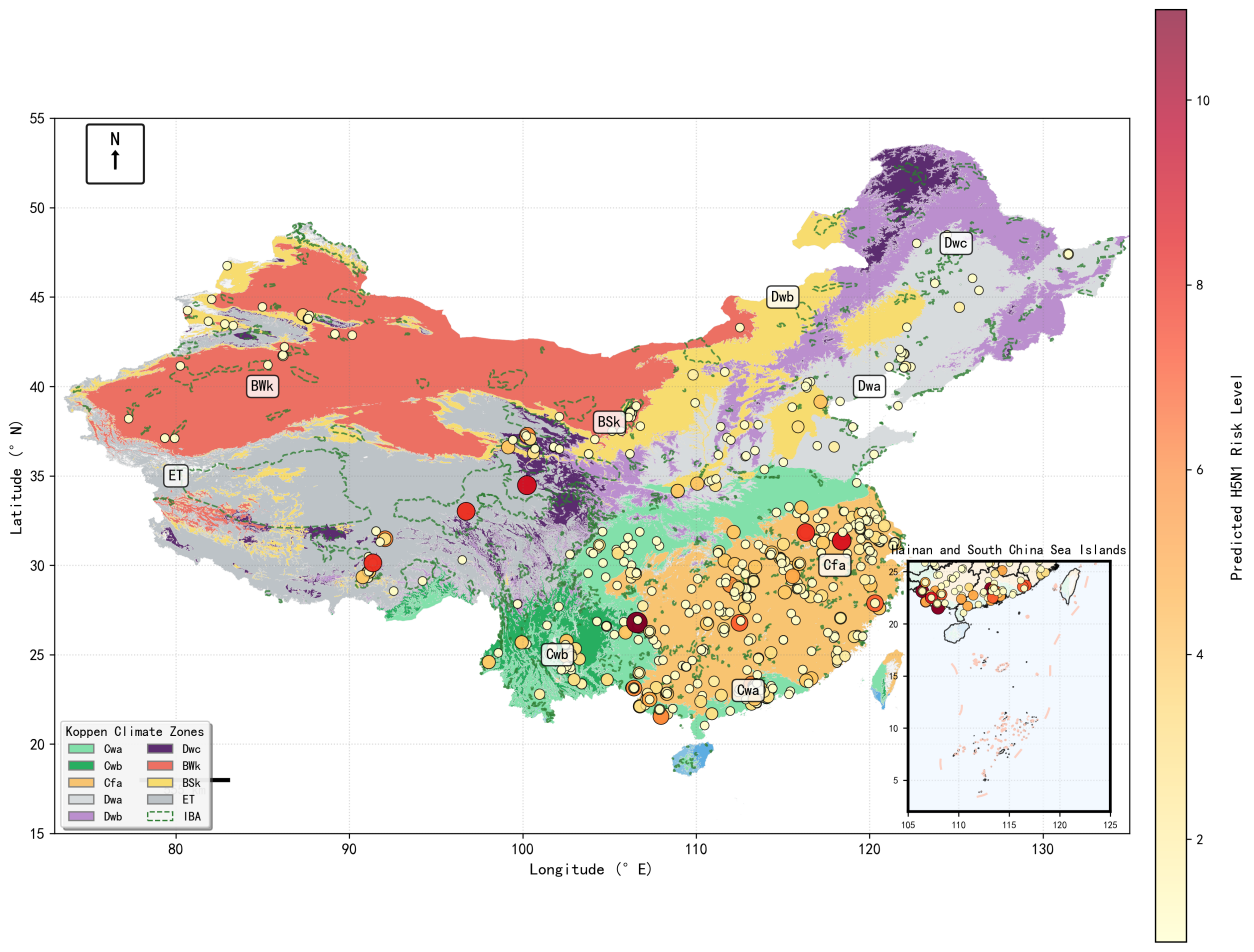
Spatiotemporal distribution of H5 avian influenza outbreaks in China, 2000–2023: H5 avian influenza risk distribution across Köppen climate zones in China.

**Figure 2:**
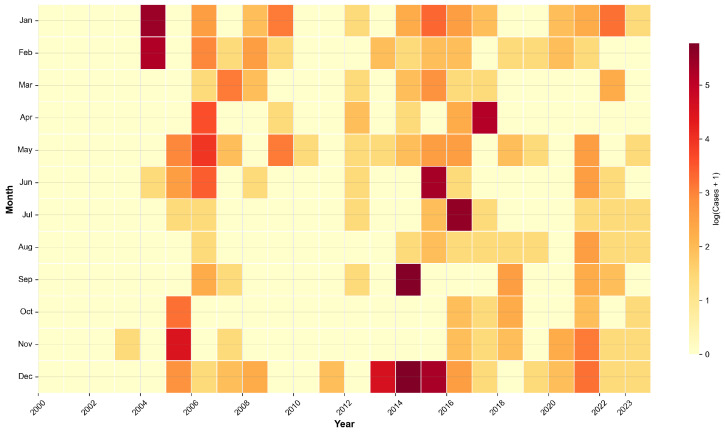
Temporal distribution of H5 avian influenza outbreaks, 2000–2023.

A total of 1800 H5 subtype avian influenza outbreaks were recorded in mainland China during the study period (Table 3). The spatiotemporal distribution of these events was highly heterogeneous. Spatially, outbreaks were predominantly concentrated in the eastern and southern provinces, particularly within the humid subtropical (Cfa) and monsoon-influenced humid subtropical (Cwa) Köppen climate zones (Figure 1). Temporally, the outbreaks exhibited strong seasonality, with incidence peaking consistently during winter and spring (December–March), and showed significant inter-annual variability with distinct epidemic waves apparent during 2004–2006 and 2014–2017 (Figure 2).

An examination of the outbreak characteristics in Table 3 reveals that the number of cases per event was highly right-skewed, with a median of 1 but a mean of 5.4 (SD = 10.2), indicating that most reported events were small despite occasional large-scale outbreaks (max = 108). The outbreaks predominantly occurred under cool conditions, with a mean ambient temperature of 7.5 °C (interquartile range [IQR]: −0.3 to 15.6 °C). Furthermore, a clear spatial relationship with wild bird habitats was evident, with 50% of outbreaks occurring within 41.2 km of an Important Bird and Biodiversity Area (IBA) and 75% occurring within 85.3 km.

### 3.2. Predictive Performance

The predictive performance and generalization ability of the final XGBoost model were robustly evaluated through a 5-fold cross-validation scheme [27]. The model demonstrated a high degree of predictive accuracy, achieving a mean coefficient of determination ($R^{2}$) of 0.776, indicating that the selected predictors explained approximately 77.6% of the variance in H5 AI outbreaks (Table 4). Notably, the $R^{2}$ score for each of the five folds consistently exceeded the performance target of 0.7.

**Figure 3:**
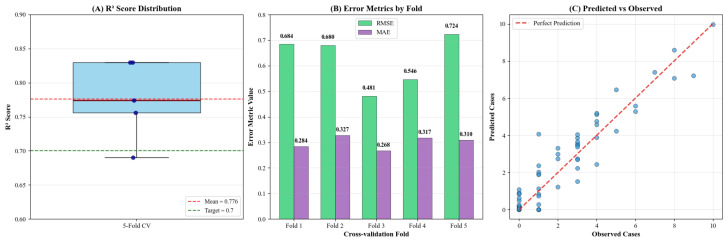
Model performance evaluation from 5-fold cross-validation.

The model’s stability was underscored by the low standard deviation of the R^2^ scores across the folds (SD = 0.065) and was visually confirmed by the narrow interquartile range in the boxplot (Table 4, Figure 3A). In terms of prediction error, the model yielded low mean values for both the root mean square error (RMSE) of 0.604 and the mean absolute error (MAE) of 0.306, which remained consistently low across all individual folds (Table 4, Figure 3B). Furthermore, rigorous overfitting controls employed during the hyperparameter optimization process resulted in an average training-test R^2^ gap of only 0.082, confirming the model’s excellent generalization capability. A visual inspection of the cross-validated predicted versus observed values is expected to further confirm this high performance, with points clustering tightly around the line of perfect agreement (Figure 3C).

### 3.3. Identification of Key Drivers of Avian Influenza Risk

**Figure 4:**
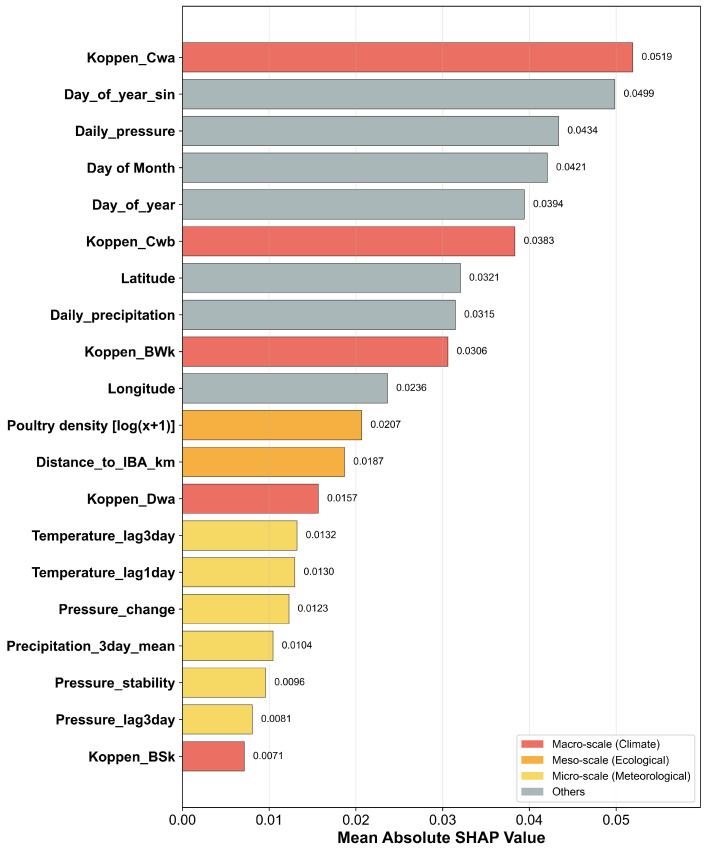
Global feature importance of avian influenza risk drivers.

The hierarchy of risk drivers for H5 avian influenza was identified by ranking all predictors based on their mean absolute SHAP values, which quantify their overall contribution to the XGBoost model’s predictions (Figure 4). The analysis revealed a clear hierarchical structure, where a small number of macro-scale contextual and seasonal factors emerged as the dominant drivers of risk [5,6]. The Köppen_Cwa (monsoon-influenced humid subtropical) climate zone was the single most influential predictor (mean |SHAP| = 0.052), followed closely by the primary seasonality feature, Day_of_year_sin (mean |SHAP| = 0.050). Other features representing the long-term environmental context and annual cycles, such as Daily_pressure, Day_of_year, and other Köppen classifications (Köppen_Cwb, Köppen_BWk), also ranked among the top predictors. This underscores the foundational role of the baseline geographical landscape and strong seasonal periodicities in determining an area’s endemic risk level.

While the macro-scale context sets the stage, meso-scale ecological factors and micro-scale meteorological variables act as crucial secondary and modulating influences (Figure 4). Key meso-scale factors related to host and wild bird interfaces, including Poultry_density (mean |SHAP| = 0.021) and Distance_to_IBA_km (mean |SHAP| = 0.019), were identified as significant predictors, although they had a smaller magnitude of contribution compared to the top-tier drivers. Micro-scale weather variables representing short-term environmental stress, such as lagged temperature (Temperature_lag3day, Temperature_lag1day) and indicators of atmospheric instability (Pressure_change), were also found to be important contributors to risk. This multi-scale structure suggests that while a region’s climate and the time of year establish a foundational risk level, the actual occurrence of an outbreak is triggered by the interplay of more dynamic, localized factors.

### 3.4. Nonlinear Effects and Risk Windows of Key Drivers

**Figure 5:**
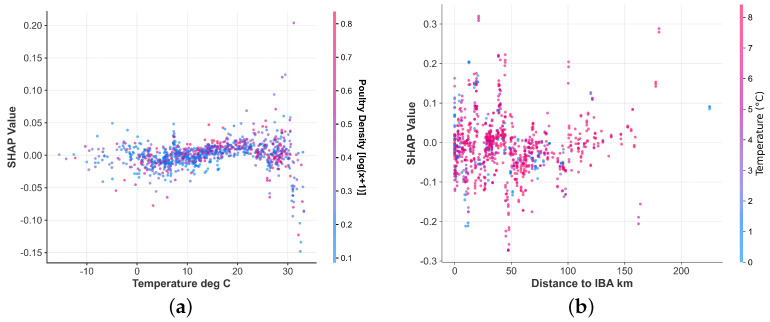
Nonlinear exposure–response curves for key predictors. (**a**) SHAP value vs. temperature; (**b**) SHAP value vs. distance to IBA.

To elucidate how the identified key drivers modulate AI risk, their marginal effects were examined using SHAP dependence plots (Figure 5). The analysis revealed clear nonlinear patterns for several key predictors, providing insights beyond simple linear correlations [32].

For ambient temperature, a distinct nonlinear trend was observed (Figure 5a). While the risk contribution remained close to zero at moderate and high temperatures (>15 °C), it began to systematically increase as temperatures fell below approximately 10 °C. This risk-enhancing effect of cold was most pronounced in the −10 °C to 0 °C range. The greater variance in SHAP values at these low temperatures suggests that the magnitude of this effect is likely dependent on other interacting factors.

Proximity to IBAs exhibited a clear negative relationship with risk (Figure 5b). The risk contribution was highest and most variable at distances less than 20 km, indicating that while close proximity is a significant risk factor, its impact is highly context-dependent. As the distance from an IBA increased, the risk contribution steadily decreased, with the effect diminishing and stabilizing beyond approximately 50–100 km.

### 3.5. Synergistic Effects Among Key Drivers

Beyond the individual nonlinear effects, our analysis delved into the synergistic interactions between predictors using SHAP interaction values. This revealed that key drivers often do not act in isolation but rather that their impacts are contingent on the context provided by other variables.

**Figure 6:**
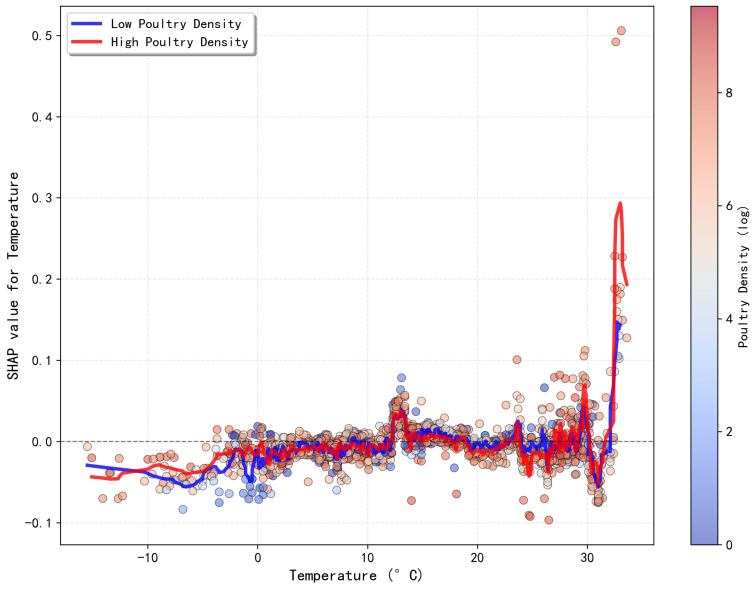
Synergistic interaction effect between ambient temperature and poultry density on H5 avian influenza risk. The plot shows the SHAP value for temperature (y-axis) across its range (x-axis), with points colored by poultry density. The clear separation of red (high-density) and blue (low-density) points at high temperatures indicates a strong positive interaction.

A particularly strong synergistic effect was identified between ambient temperature and poultry density (Figure 6), demonstrating that the influence of temperature on risk is highly dependent on host density. Specifically, within the 0–25 °C range, the marginal effect of temperature on risk was negligible regardless of poultry density. However, a powerful positive interaction emerged at temperatures above 30 °C. In these high-temperature conditions, the risk contribution from temperature in high-density scenarios increased sharply to a SHAP value approaching 0.3 while remaining near 0 in low-density settings (Figure 6). Conversely, no such synergistic amplification was observed in the low-temperature range (<0 °C). This suggests that while cold temperatures act as a general risk factor (as shown in Figure 5a), their effect is not amplified by high poultry density in the same way that high temperatures are. This finding reveals a critical mechanism: high host density acts as an amplifier, transforming otherwise low-risk, high-temperature conditions into a significant driver of H5 outbreaks.

**Figure 7:**
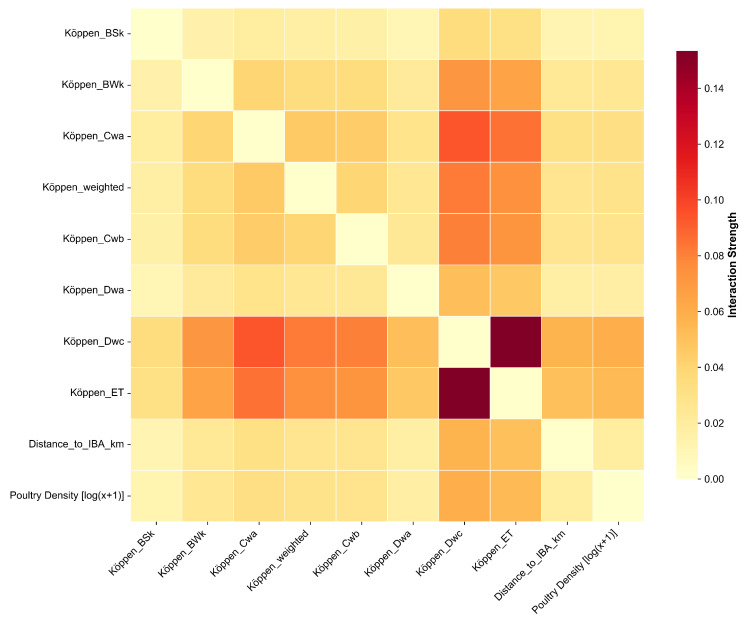
Heatmap of pairwise SHAP interaction strengths among key predictors. The color of each off-diagonal cell indicates the strength of the interaction effect between the corresponding pair of features, with brighter, redder colors signifying stronger synergies. The diagonal represents the main effect of each feature.

To provide a comprehensive overview of all pairwise interactions, a SHAP interaction heatmap was generated (Figure 7). This visualization systematically maps the interaction strength between all key predictors. The heatmap highlights several notable synergies beyond the temperature–poultry relationship. For instance, strong interactions are visible between certain Köppen climate zones, such as Köppen_Dwc and Köppen_ET, and between climate zones and ecological factors like Distance_to_IBA_km. These complex interdependencies, revealed by the heatmap, underscore the importance of a holistic, context-aware approach to risk assessment, as the impact of any single driver is often modulated by the broader environmental and ecological setting.

### 3.6. High-Resolution Spatiotemporal Risk Mapping

**Figure 8:**
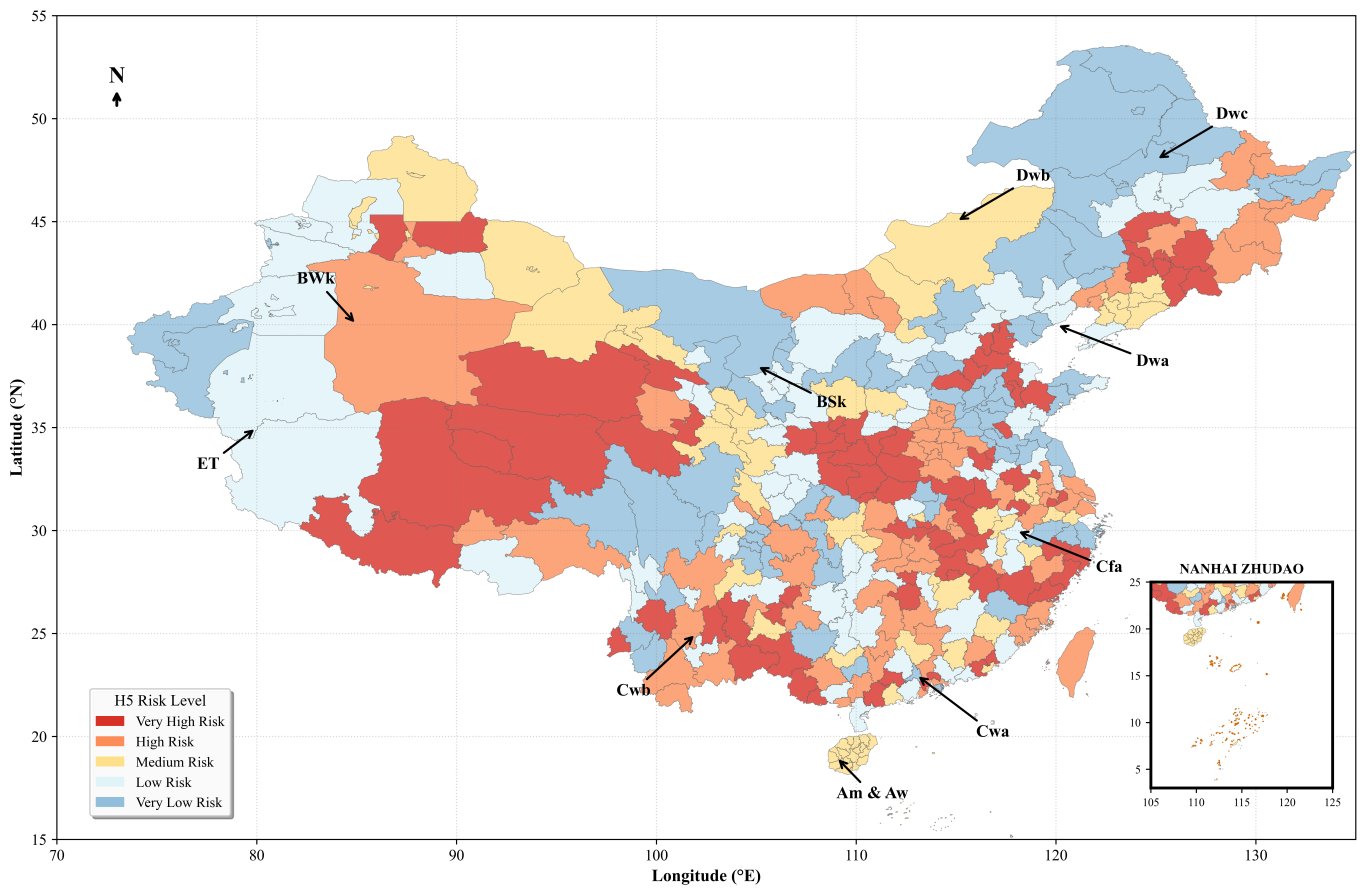
High-resolution predictive risk map of H5 avian influenza in mainland China.

To synthesize the model’s findings into a practical decision-support tool, the trained XGBoost model was applied to a high-resolution nationwide grid, generating a predictive baseline risk map for H5 avian influenza across mainland China (Figure 8). The map reveals a distinct and highly heterogeneous geographical distribution of risk. High-risk areas are prominently concentrated in eastern and southern China, forming several key hotspots. These include the Yangtze River Delta, the Pearl River Delta, the Sichuan Basin, and the regions surrounding major lake systems such as Poyang Lake and Dongting Lake.

The spatial patterns delineated on the risk map show strong correspondence with the key drivers identified in the feature importance analysis. The predicted high-risk zones largely overlap with areas characterized by a convergence of multiple top-ranked risk factors: high poultry density, humid temperate/subtropical climate conditions (e.g., the Cfa and Cwa Köppen zones), and proximity to IBAs (cf. Figure 4) [21,33]. Conversely, western and northern regions, such as the Tibetan Plateau and the arid areas of Xinjiang and Inner Mongolia, which lack this combination of risk factors, consistently exhibit the lowest predicted risk levels. The map therefore serves as a visual confirmation of the hierarchical and interactive driver structure identified by the model.

## 4. Discussion

This study applied an interpretable machine learning framework to assess the spatiotemporal risk of HPAI H5 avian influenza in mainland China. While previous studies have provided significant insights into HPAI dynamics, understanding the complex, multi-scale interplay of drivers across large, heterogeneous areas remains a challenging area of research [34]. Our work contributes to this field by analyzing over two decades of dynamic, multi-source data with an XGBoost-SHAP framework. This approach yielded a model with strong predictive performance and, more importantly, offered a way to explore the hierarchical, nonlinear, and synergistic relationships that may govern disease risk. The analysis suggests a potential hierarchical structure of risk drivers, explores nonlinear exposure–response patterns, and identifies possible synergistic effects. These findings were synthesized into a high-resolution risk map, which may offer…which may offer valuable insights for the planning of surveillance and control activities.

### 4.1. Advantages of the Study

A primary finding from our model is the apparent importance of the macro-scale ecological landscape in shaping HPAI H5 risk [35]. Our analysis identified poultry density and specific Köppen climate zones as predictors with high feature importance, suggesting that the baseline risk of an outbreak may be strongly influenced by the geographical and agricultural context [21]. This observation is consistent with a substantial body of literature that has frequently identified high poultry density as a key factor in HPAI H5N1 amplification and spread in various settings [23]. Our work contributes to this by illustrating how this baseline risk appears to be modulated by meso-scale factors, such as the interface with wild birds proxied by IBAs [24], and potentially triggered by micro-scale meteorological events. A significant aspect of our study is the characterization of complex, nonlinear relationships. For instance, our model identified a distinct nonlinear relationship between ambient temperature and AI risk (Figure 5a). The risk contribution remained negligible at moderate to high temperatures but increased substantially as temperatures dropped into colder ranges (e.g., below 10 °C). This finding aligns with existing laboratory and ecological evidence on enhanced viral survival and stability in cold conditions [26,36]. Similarly, the non-monotonic effect of poultry density suggested by our analysis, which indicates a potential plateauing of risk at very high densities, provides a data-driven hypothesis that moves beyond a simple “more is worse” assumption. Such nonlinear insights, if further explored, could be valuable for refining targeted and resource-efficient control strategies. The comprehensive interaction heatmap (Figure 7) further revealed a landscape of complex interdependencies, such as those between different climate zones and ecological factors, underscoring the necessity of a context-aware approach to risk assessment.

Furthermore, our analysis suggests that these drivers may not operate in isolation but could exhibit significant synergistic effects, a concept of growing importance in ecological modeling [37]. A notable interaction identified by our model was between ambient temperature and poultry density (Figure 6). The results point towards a potential compound risk mechanism: high temperatures (e.g., >30 °C), which independently show a negligible association with risk, may act as a significant risk amplifier when they co-occur with high poultry density. This observation, if validated by further mechanistic or field studies, could be particularly important as it might challenge conventional assumptions about risk seasonality in certain high-density poultry farming systems [38,39]. It suggests a testable hypothesis that during summer months, surveillance resources could be more efficiently allocated by prioritizing regions with a high concentration of poultry. This ability to uncover and quantify potential interactions is a key feature of our interpretable machine learning approach.

### 4.2. Limitations

Nevertheless, our study is subject to several limitations that warrant acknowledgment. First, as an ecological study, its findings are subject to the ecological fallacy, and associations observed at a regional scale may not directly translate to individual farms. Second, the outbreak data, while being the best available, may be influenced by regional variations in surveillance intensity and reporting practices, a common challenge in large-scale epidemiological analyses [40,41,42]. Third, the predictor variables, while comprehensive, serve as proxies for more complex processes; for instance, “distance to IBA” is a simplification of intricate wild bird migratory patterns.

### 4.3. Future Perspectives

These limitations highlight valuable avenues for future research. A logical next step could involve integrating a phylodynamic analysis of viral genomic data to trace transmission pathways with greater precision. Moreover, incorporating more dynamic data layers, such as poultry trade networks or real-time migratory bird tracking data, could further refine the model’s predictive capabilities. Our aggregation of HPAI H5 subtypes, while necessary for this analysis, also points to the need for future subtype-specific risk modeling (e.g., for H5N1 vs. H5N6) as more granular data become available [43,44]. Finally, applying this validated modeling framework to future climate and land-use change scenarios could provide important insights for long-term preparedness and policy planning [45].

## 5. Conclusions

In conclusion, this study reveals that the spatiotemporal risk of H5 avian influenza in China is governed by a distinct hierarchy of multi-scale ecological drivers. The final interpretable machine learning model achieved a high degree of predictive accuracy (5-fold CV R^2^ = 0.776), demonstrating that stable, macro-scale contexts, such as regional climate zones and poultry density, provide the foundational layer of risk, which is then modulated by transient, micro-scale meteorological triggers. Critically, the analysis moved beyond identifying linear risk factors to uncover a novel synergistic interaction: high ambient temperatures (>30 °C), typically considered low-risk, become a significant risk amplifier in areas with high poultry density. By synthesizing these hierarchical, nonlinear, and synergistic effects into a high-resolution risk map, this work provides an evidence-based tool for developing more targeted surveillance and control strategies. Future research should build upon this framework by integrating more dynamic variables, such as viral genomic data and specific intervention measures, to further enhance predictive accuracy and response planning [26,32,46,47].

## Figures and Tables

**Table 1:** Description and justification of predictor variable categories.

Scale	Category and Variables	Justification and Supporting References
Macro-scale	Geographic and Climatic Contexts (e.g., latitude, longitude, Köppen class)	Represents stable, large-scale environmental conditions that define baseline AI risk. The Köppen–Geiger classification summarizes long-term climate regimes governing viral persistence [21,23].
Meso-scale	Host and Wild Bird Interface (e.g., poultry density, dist. to IBA)	Captures key ecological factors at a regional level. Poultry density is a fundamental determinant of disease amplification, while proximity to IBAs proxies for viral spillover risk from wild birds [23,24,25].
Micro-scale	Meteorological Triggers (e.g., lagged temp., pressure change)	Represents transient weather conditions that can trigger outbreaks. Temperature and other factors influence the environmental survival and stability of the influenza virus [23,26].
Temporal	Seasonality Controls (e.g., sine/cosine of day of year)	Models the well-documented seasonality of avian influenza. These cyclical features allow the model to learn patterns of risk peaking in cooler months in a continuous manner [4].

**Table 2:** Comparative performance of the XGBoost model against traditional statistical models. All models were evaluated using a 5-fold cross-validation scheme on the same set of predictors. Metrics shown are the mean and standard deviation (SD) across the folds.

Model	Mean R^2^ (±SD)	Mean RMSE (±SD)	Mean MAE (±SD)
XGBoost (this study)	0.776 (±0.039)	0.604 (±0.065)	0.306 (±0.060)
PanelOLS (fixed effects)	0.458 (±0.052)	0.881 (±0.095)	0.573 (±0.048)
Gaussian GLM	0.257 (±0.061)	0.995 (±0.112)	0.634 (±0.055)

**Table 3:** Descriptive statistics for all variables used in the model.

Variable	N	Mean	Std. Dev.	Median	Min	Max	Q25	Q75
Cases	1800	5.4	10.2	1	1	108	1	2
Mean temperature (°C)	1800	7.5	10.1	14.57	−14.5	34.73	−0.3	15.6
Atmospheric pressure (hPa)	1800	955.74	95.17	997.75	572.95	1038.9	922.27	1012.55
Precipitation (mm)	1800	2.33	9.12	0	0	145.8	0	0.1
Lagged temperature (°C)	1800	14.71	10.25	14	−15.5	33.6	6.97	24.2
Lagged pressure (hPa)	1800	952.02	109.96	998.9	0	1038.8	922.15	1011.5
Lagged precipitation (mm)	1800	5.57	109.22	0	0	3272	0	0.2
Poultry density (log-transformed)	1800	5.95	2.28	6.55	0	9.53	5.32	7.45
Distance to IBA (km)	1800	44.8	37.4	41.2	0	225	16.9	85.3
Within IBA (binary)	1800	0.053	0.225	0	0	1	0	0
Longitude (°E)	1800	110.05	8.98	112.22	77.27	131.47	106.41	115.97
Latitude (°N)	1800	30	6	29	21	48	25	32
Month (sin-transformed)	1800	−0.005	0.615	0	−1	1	−0.5	0.5
Month (cos-transformed)	1800	0.202	0.763	0.5	−1	1	−0.5	0.866
Day of year (sin-transformed)	1800	−0.041	0.604	0	−1	1	−0.538	0.448
Day of year (cos-transformed)	1800	0.208	0.768	0.556	−1	1	−0.374	0.962
Köppen 14.0 (binary)	1800	0.475	0.5	0	0	1	0	1
Köppen 11.0 (binary)	1800	0.255	0.436	0	0	1	0	1
Köppen 7.0 (binary)	1800	0.07	0.255	0	0	1	0	0

Std. Dev.: standard deviation; Q25/Q75: 25th and 75th percentiles; IBA: Important Bird and Biodiversity Area. The Köppen variables are one-hot encoded representations of specific climate zones.

**Table 4:** Model performance metrics from 5-fold cross-validation.

Metric	Mean	Std. Dev.
$R^{2}$	0.776	0.039
RMSE	0.61	0.07
MAE	0.318	0.009

## Data Availability

The raw H5 outbreak data analyzed during the current study are publicly available from the Food and Agriculture Organization’s Global Animal Disease Information System (EMPRES-i). The processed data and the final set of predictor variables generated during this study are available from the corresponding author on reasonable request.

## Abbreviations

The following abbreviations are used in this manuscript:

AI	Avian Influenza
HPAI	Highly Pathogenic Avian Influenza
FAO	Food and Agriculture Organization
EMPRES-i	Global Animal Disease Information System
CMA	China Meteorological Administration
GLW	Gridded Livestock of the World
IBA	Important Bird and Biodiversity Area
XGBoost	Extreme Gradient Boosting
SHAP	SHapley Additive exPlanations
CV	Cross-Validation
RMSE	Root Mean Square Error
MAE	Mean Absolute Error
VIF	Variance Inflation Factor

## Appendix A. Model Hyperparameters

The final optimized hyperparameters for the XGBoost model, determined through a 5-fold cross-validation and tuning process, are detailed in Table A1.

**Table A1:** Final optimized hyperparameters for the XGBoost model.

Hyperparameter	Final Value
n_estimators	750
max_depth	4
learning_rate	0.0875
min_child_weight	2
subsample	0.9106
colsample_bytree	0.8703
reg_alpha	0.2717
reg_lambda	0.7399
gamma	0.0034
scale_pos_weight	0.975
max_delta_step	0
objective	‘count:poisson’
random_state	42
n_jobs	1
